# Monitoring and Management of Antipsychotic-Induced Hyperprolactinaemia With a Focus on Bone Health

**DOI:** 10.1192/bjo.2026.11756

**Published:** 2026-06-30

**Authors:** Ahmad Yafawi

**Affiliations:** Merseycare NHS trust/Warrington and Halton Teaching Hospital, Liverpool / Warrington, United Kingdom

## Abstract

**Aims:**

Antipsychotic-induced hyperprolactinaemia is a common adverse effect associated with dopamine-blocking medications. Persistently raised prolactin is linked to sexual dysfunction, infertility, and importantly, reduced bone mineral density and osteoporosis. Concerns were raised that prolactin monitoring within the service was inconsistent and that raised levels were not always acted upon, posing potential long-term risks.

**Aims:**

- Assess whether patients prescribed antipsychotics had prolactin levels appropriately monitored, during my psychiatry placement at the Early Intervention team in Adult psychiatry.
- Determine whether elevated prolactin prompted clinical action.
- Identify gaps in monitoring practice and associated risks to bone health.
- Provide recommendations to improve standards of care.

**Methods:**

A retrospective audit of referrals over a six-month period was performed usingRiOelectronic records.

- Patients **not** on antipsychotics were **excluded**.
- For eligible patients, the following were reviewed:
  1. Antipsychotic medication.
  2. Whether prolactin was checked.
  3. Whether levels were normal or raised.
  4. Documentation of any medication adjustment or follow-up.
- Comments were added to highlight missed monitoring opportunities or incomplete documentation.

**Results:**

- **Total referrals reviewed:** 43
- **Excluded (not on antipsychotics):** 20
- **Included for audit:** 23

**Key Findings:**

- **6/23 (26%)**had*no prolactin test* despite being on antipsychotics.
- **15/23 (65%)** had *normal prolactin* results.
- **2/23 (9%)** had *raised prolactin*, with one severe elevation (4155.6 mU/L).

Only **1 patient** had clear medication adjustment in response to raised prolactin

**Implications for Bone Health:**

- Persistent or unmonitored hyperprolactinaemia increases osteoporosis risk.
- No consistent documentation linking raised prolactin to bone-health outcomes (e.g., DEXA, vitamin D).

**Conclusion:**

Overall, the team demonstrates good awareness and management of prolactin-related issues, with most patients receiving appropriate monitoring and normal results. When significantly raised prolactin levels were identified, clinicians acted promptly with medication review and plans for follow-up, reflecting good clinical practice.

The key learning point is the value of maintaining this strong standard while improving documentation clarity and ensuring timely follow-up of repeat prolactin testing. No major pathway changes are required; instead, small refinements can further strengthen already effective care.

The QIP identified only one patient with raised prolactin who had not yet received a medication adjustment.

